# Beyond Genetics—Stratified and Personalised Medicines Using Multiple Parameters

**DOI:** 10.3390/ph3051637

**Published:** 2010-05-25

**Authors:** Richard Peck, Patrick Smith

**Affiliations:** 1 Roche Products Ltd., Hexagon Place, 6 Falcon Way, Shire Park, Welwyn Garden City, AL7 1TW, UK; 2 Roche Palo Alto, LLC., 3431 Hillview Ave, Palo Alto, CA 94143, USA; E-Mail: Patrick.smith@roche.com

**Keywords:** pharmacogenetics, modeling, response variability, dose-finding

## Abstract

Prescribers have been practicing stratified medicine for many years. Patient characteristics, usually non-genetic, including age, comorbidities and concomitant medications are taken into account when deciding which drug to prescribe. In addition, the majority of drugs require dose adjustments across patient subgroups, usually determined by non-genetic differences between the subgroups. Whilst pharmacogenetics hold promise for enhancing treatment stratification and even treatment individualisation, non-genetic factors will continue to be very important. Both non-genetic and genetic factors must be considered to improve understanding and quantification of the variability in treatment outcomes and to guide stratification and targeting of patient subgroups to the right drug and also to the right range of doses within that subgroup. Development of stratified medicines must consider non-genetic as well as genetic factors and, where appropriate, include stratification through optimising the dose for each patient or subgroup as well as by choosing the drug most likely to deliver efficacy to that patient or group.

## 1. Introduction

Personalised or stratified medicine and healthcare is often thought of in terms of developing tests, usually genetic, to distinguish the patient subgroups, or even individual patients, who will respond to a drug from those who will not. Or stated alternatively, to identify the conditions under which an individual will have an optimal response. The US Department of Health and Human Services defines it as: “Personalised health care describes medical practices that are targeted to individuals based on their specific genetic code in order to provide a tailored approach. These practices use preventive, diagnostic, and therapeutic interventions that are based on genetic tests and family history information. The goal of personalised health care is to improve health outcomes and the health care delivery system, as well as the quality of life of patients everywhere” (http://www.hhs.gov/myhealthcare/glossary/glossary.html/). In the case of efficacy the concept is to identify responders who should receive the drug and in the case of safety to identify those who should not. However, there are response identifiers other than genetic tests that can allow identification of the responder groups or a combination of results from several tests will be required to make good predictions. For example, a warfarin dose optimisation protocol that combines both non-genetic and genetic factors is more predictive of eventual warfarin dose than one based on clinical factors alone [1]. An alternative mantra is the phrase “Right patient, right drug, right dose”, which introduces the additional idea that, amongst the subgroup capable of responding to the drug, different patients may still require different dose regimens in order to obtain the desired response.

Trusheim and colleagues have proposed a model of personalised medicine as a continuum between empiric medications, which work for almost all patients with little individualisation needed, to stratified medicines, and individualised medicines. A stratified medicine is the next level of individualisation, and can be defined as those medicines which work for a subset of the population based on a genetic test or biomarker, such as breast cancer patients who are her-2 positive being treated with trastuzumab. Individualised medicine is the extreme example of a medicine, such as a vaccine, that is specifically manufactured for an individual patient. For the purposes of this article, personalised medicine can be considered the tailoring of treatment to a particular individual based on numerous characteristics which influence drug response. In this context, even a stratified medicine has the potential to be personalised by further optimising the dose based on other patient characteristics which influence pharmacokinetic or pharmacodynamic response [2].

## 2. Sources of Variability

**Figure 1:**
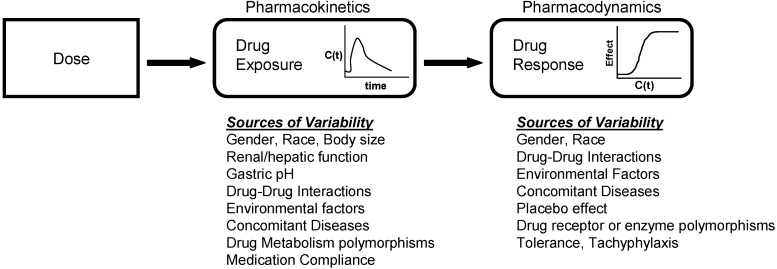
The relationship between dose and clinical effect. Drug exposures, C(t), determine where a given individual is on their own unique concentration-effect curve. Each individual will have one pharmacokinetic profile, and numerous pharmacodynamic profiles for on target and off-target effects. Sources of variability in both pharmacokinetics and pharmacodynamics which contribute to differences in response to drugs are listed.

At the core of clinical pharmacology is the understanding of the relationship between dose, exposure and effects of drugs. Much of a clinical pharmacologist’s work is investigating and understanding the sources of variability in these relationships to help identify in which patients the drug is most likely to have efficacy, the dose or doses to be used and how to adjust doses in order to maintain efficacy and safety in different patient groups. Variability in response can be due to variability in the relationship between dose and exposure [pharmacokinetic (PK) variability] or between exposure and effect(s) [pharmacodynamic (PD) variability]. The distinction between PK and PD variability is somewhat arbitrary but it is a convenient simplification. It is paramount to recognize that the link between a given dosage regimen and therapeutic outcome is driven by both pharmacokinetics and pharmacodynamics. When a drug is administered to a patient, the individual pharmacokinetics will determine the concentration of the drug at the site of action for both safety and efficacy. The amount of drug at the site of action then elicits a particular pharmacodynamic response, which is governed by how ‘sensitive’ that patient is to the drug. Clinical response is therefore a function of the link between dose, pharmacokinetics, and pharmacodynamics. To optimize drug therapy, it is necessary to consider all sources of variability in this chain, so that the appropriate dose of the appropriate drug can be administered. This concept is illustrated in Figure 1. 

PK variability is related to many factors, including age, sex, ethnicity, genetics of metabolic enzymes and transporters, diet, smoking habits, organ function, disease state, concomitant medications and patient compliance. Typically PK variability is well recognised and extensively investigated during drug development to generate prescribing information that stratifies patients into groups requiring different doses to achieve the same exposures. It is obvious that genetics plays an important role in understanding and potentially controlling for the variability in the relationship between dose and exposure, but there are many other factors which may often be more important. Usually, variability in PK constitutes a minor proportion of the total variability in response compared to variability in PD [3]. However, for some drugs, PK variability may be the driving factor determining successful clinical outcomes or resulting in severe adverse effects.

PD variability in the relationship between exposure and effect may be due to heterogeneity in the underlying disease, differences in the interaction of the drug with its molecular target or in the downstream consequences of the effect at the molecular target, the effects of concomitant medication (other than on drug exposures), or the impact of comorbidities. Trastuzumab is perhaps the archetype of a drug whose activity is related to disease heterogeneity, patients with tumours that do not over-express her-2 neu do not respond to trastuzumab regardless of the dose administered. But, in general, although variability in exposure effect relationships is well recognised, it is less well understood than the dose/exposure variability. 

## 3. Clinical Impact of Variability

It is widely recognised that most therapies do not work in all, or even the majority of patients [4]. This is despite the widespread use of dose adjustments to account for common causes of variability in exposure, including age, organ dysfunction and the use of concomitant medication. Given the multifactorial complexity of human diseases and drug therapy, it seems unlikely that one simple genetic test will usually be sufficient to explain 100% of the clinical response variability. While there are examples where this is the case, they are relatively rare given the thousands of marketed drugs available in clinical practice. A single test may frequently be unable to give the health care provider sufficient information to select the drug and dose for a given patient with the highest probability of treatment success and a low probability of toxicity. In patients with tumours over-expressing her-2, the addition of trastuzumab therapy to doxorubicin/cyclophosphamide/taxotere in adjuvant breast cancer increases the one year disease free survival rate from 7.5% to 13%, with a small increased risk of cardiac toxicity [5]. Despite this clinically significant 50% reduction in cancer recurrence rate, might it be possible to further individualise this stratified medicine while reducing patients at risk for cardiotoxicity ? 

Approximately 50% of patients with chronic genotype 1 hepatitis C infection fail to achieve cure with the standard of care therapy, pegylated-interferon and ribavirin. This very large dichotomy in response remained elusive until recently when polymorphisms of the IL28B gene were identified which are associated with clinical response [6,7,8] Patients homozygous for wild type IL28B have approximately an 80% cure rate with interferon based therapy, whereas individuals either heterozygous or homozygous for a genetic polymorphism have approximately a 25% and 35% cure rate, respectively [6]. While this genetic test explains a substantial proportion of the variability in interferon response, much of the response variability is yet to be identified as genetic-related, with factors such as body size, ethnicity, viral load and viral genotype still accounting for a significant proportion of the variability in response. Without also incorporating non-genetic factors into a treatment algorithm, it may be unlikely that an IL28B genetic test alone would prove to be particularly useful in clinical practice. However, incorporation of this important genetic test with other non-genetic factors may significantly improve the ability to successfully treat patients with chronic hepatitis C infection.

Personalised medicine holds the promise to produce optimal efficacy with minimal side effects in every patient. Achieving this result can only be accomplished through the control of both inter- and intra-subject variability. 

The clinical relevance of variability in drug response depends on the magnitude of the variability, its source and potential for modification, and the therapeutic index of the drug. For a drug with a large therapeutic index and little risk of troublesome side effects, it is perfectly reasonable to use a high dose in all patients to ensure maximum efficacy in the population. In this case, overdosing some individuals does not increase the risk of adverse effects, but ensures that the patients who are less sensitive to the drug receive the adequate exposures to result in efficacy. Examples of such medications include penicillin antibiotics, where a large safety margin exists, and doses are selected to ensure plasma levels remain sufficiently high to exceed the minimum inhibitory concentrations of common pathogens [9].

However, for a drug with a narrow therapeutic index, using a high dose is not a reasonable solution as it would place a population of patients at undue risk of adverse events. Therefore, the traditional approach in drug development for narrow therapeutic index drugs is to select a lower dose for the population at large, which avoids troublesome or serious side effects, but also results in certain patients being under-dosed and therefore not receiving a positive clinical outcome from therapy. This approach is successful if a particular drug can be initiated at a low dose and slowly titrated to the desired effect. In this manner, the clinician attempts to define the individual’s personal therapeutic index through the process of trial and error. It may take some time to identify a therapeutic dose, which may potentially place the patient at risk for disease progression or side effects. Classic examples of drugs which are utilised in this manner include insulin, oral hypoglycaemic drugs and warfarin. 

However, despite improvements in predicting warfarin dosing through genetic testing, an even safer and more effective way of optimising warfarin doses is desirable. The same would likely be true for optimising treatments for chronic conditions—a faster onset of benefit, avoiding adverse effects, fewer follow up appointments and/or tests to adjust therapy, and greater convenience for patients and physicians. The ability to have predefined predictors of dose, potentially through the use of genetic tests and/or mathematical modelling which incorporates known genetic and predictive factors (see below), could significantly improve the ability to get individual patients to therapeutic levels more rapidly. 

## 4. Controlling Variability in Drug Response

**Figure 2:**
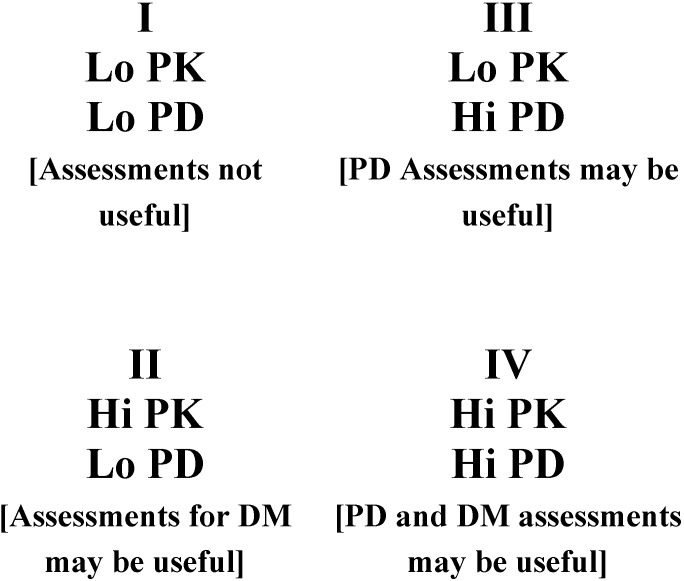
Pharmacokinetic/Pharmacodynamic variability and the utility of assessing a patient to individualise therapy. Therapies falling into quadrant I are unlikely to benefit from phenotypic or genotypic testing; Quadrants II and IV may benefit from a pharmacokinetic assessment (genetic or non-genetic) of drug metabolising enzymes or transporters; Quadrants III and IV may benefit from a pharmacodynamic assessment (genetic or non-genetic) to identify likelihood of clinical response. Controlling variability is more useful for drugs with a narrow therapeutic index. DM = Drug Metabolism/transporters; PK = Pharmacokinetics; PD = Pharmacodynamics.

The clinical benefit of controlling variability will depend upon the drug and its specific risk benefit ratio. In clinical practice, the utility of a particular genetic or non-genetic test for either pharmacokinetics or pharmacodynamics will depend not only on its positive and negative predictive value, but also on the source and magnitude of variability of a given drug. Figure 2 illustrates examples of the magnitude and source of variability and the associated utility of both pharmacokinetic and/or pharmacodynamic tests. In cases where variability is small, there is little utility in attempting to control. If drug metabolising enzymes or transporter variability plays a large role, then individualizing doses based on an assessment of a patient’s drug metabolizing enzyme activity or transporters may generate useful information that can result in dose individualisation. If there is large variability in pharmacodynamic response, then making an attempt to control for pharmacodynamic variability either through an assessment of phenotypic response or a pharmacogenomic test, is likely to be useful in identifying patients who may or may not respond to a drug or who may require doses which differ from the typical population.

Drugs falling into Quadrant I (Figure 2) are the easiest to utilise in the clinical setting, as variability is low and most patients will exhibit a favourable response at the same dose. Therefore, the “one dose for all” approach will work well. Quadrant II represents drugs where most patients will respond favourably to treatment at a given exposure level, but variability in PK necessitates dose individualisation to achieve this desired exposure target. Quadrant III includes medications where the variability in PK is low, but the high variability in PD requires dose individualisation to identify the proper dose to elicit the desired treatment response. Quadrant III drugs would benefit from an assessment of PD variability, while there would be little reason to attempt to control for variability in PK. Quadrant IV drugs include agents with high variability in PK and PD, and are the most difficult to use in clinical practice without the ability to assess and control for variability in PK and/or PD, particularly if such an agent has a narrow therapeutic index.

There are an increasing number of examples of drugs that are prescribed, typically at a fixed dose or one adjusted based only on body weight, only after undertaking a test, typically genetic, to confirm either the likelihood of response (e.g., trastuzumab, imatinib) or the absence of a predictor of non-response (e.g., anti-EGFR antibodies and k-ras mutations) or the absence of a predictor of adverse effects (e.g., abacavir, irinotecan). And stratification of patients, and doses, to compensate for factors altering the relationship between dose and exposure is well established in drug development and use. However, despite the large amount of residual variation in response, there are relatively few examples of drug doses that are adjusted for reasons other than optimising exposure, although such dose optimisation is feasible and can be useful. 

**Figure 3:**
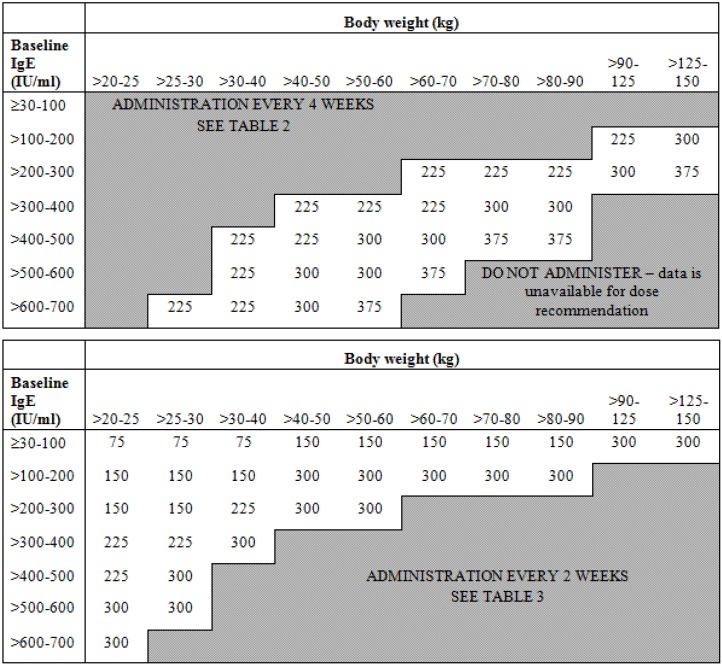
Omalizumab dosing algorithm.

Early in the development of the anti-IgE monoclonal antibody, omalizumab, it was recognised that response was associated with baseline IgE concentrations. Initial studies demonstrated a relationship between baseline IgE levels and the dose required to decrease IgE below 50 ng/mL and between baseline IgE level, dose, and activity in allergic rhinitis [10,11]. In consequence a dosing algorithm was developed (Figure 3) to take account of body weight and baseline IgE concentrations in order to give a reduction in IgE levels below 50 ng/mL. These doses were confirmed to be efficacious in patients with asthma and increased IgE [12,13]. Recent PK/PD studies have confirmed the importance of dosing omalizumab to maintain IgE concentrations below 50 ng/mL in order to maintain efficacy in asthma [14]. 

In the treatment of HIV with antiretrovirals, it is recognised that maintaining sufficiently high concentrations of many drug classes, which exceeds the minimum concentration needed to inhibit viral replication, is necessary at all times to prevent therapeutic failure and resistance. With most antiretrovirals, there is large variability in PK, which is taken into consideration in drug development to select doses based on this paradigm. Further, in clinical practice, individualisation of dosing may be accomplished via a determination of a patient’s pharmacokinetics and the susceptibility (phenotype and/or genotype) of the virus infecting this particular patient. Incorporating both individualised PK and PD at the patient level is used to modify treatment, to ensure that the minimum plasma concentration at the end of the dosing interval exceeds the viral IC_50_ [15]. While large prospective trials using this approach in HIV are limited, current treatment guidelines suggest that such an approach may be beneficial for specific patient populations, such as those that are treatment experienced or are expected to have altered pharmacokinetics [16]. Similar approaches to dosing based on pathogen susceptibility is well recognised in antibiotic therapy. As long as the drug exposures in an individual patient sufficiently exceed the pathogen susceptibility target, excellent clinical outcomes are observed [9]. In other words, more susceptible pathogens can be sufficiently treated with lower doses, whereas more resistant pathogens require higher doses, but achieve similar clinical outcomes.

Genetic polymorphisms might not always be viewed as a yes or no determination with regard to the use of a particular drug in a patient, but rather to define the conditions under which the drug can be administered safely and effectively. For example, thiopurine *S*-methyl transferase (TPMT) is the primary enzyme which metabolizes 6-mercaptopurine and azathiopurine, medications used in a variety of neoplastic diseases. In patients with TPMT deficiency, standard doses of 6-mercaptopurine may result in extremely high drug concentrations and fatal haematopoietic toxicity. However, TPMT patients can be successfully treated with much lower doses of 6-mercaptopurine, with good clinical outcomes obtained with 5–10% of the standard dosages [17]. Understanding the key source of variability for 6-mercaptopurine, and then successfully controlling it with reduced doses, led to an ability to treat both TPMT wild type and deficient patients. 

There is marked variability in the platelet aggregation response to clopidogrel with some patients showing little or no change in platelet aggregation on the approved dosing regimen [18,19,20,21]. Clopidogrel is a prodrug metabolised to an active form by cytochrome P450 family enzymes including CYP3A4 and 2C19. Polymorphisms of 2C19, combined with age, BMI and plasma lipid concentrations account for 22% of the response variability [21] and variability in CYP3A4 activity probably accounts for some of the remainder [18]. There are now several clinical trials suggesting that decreased anti-platelet effect of clopidogrel is associated with reduced efficacy [21,22,23,24] whilst prasugrel, which produces an anti-platelet effect in almost all subjects [25], has demonstrated greater efficacy [26]. It is certainly possible that a dose optimisation approach, using tests of platelet aggregation to adjust clopidogrel dose to a target effect level, could produce enhanced clinical efficacy but this will require confirmation in clinical trials. Dose selection based on platelet aggregation tests as a measure of drug activity, would overcome the lack of understanding of all the factors, genetic and non-genetic, associated with impaired response and could be used to guide dose adjustments. Alternatively, those patients with an inadequate response to a test dose of clopidogrel could be prescribed prasugrel instead.

## 5. Potential for Model-Based Methods.

Mathematical modelling attempts to formally characterise a given system in a quantitative manner. Modelling consists of writing a series of mathematical equations, which captures the behaviour of a complex system, and may incorporate the sources and magnitudes of variability. A more thorough review of this topic is available from a number of sources [27,28,29]. Such approaches to modelling systems have successfully been applied in a large number of industries, including aerospace, auto design and manufacture, geology, and increasingly in the pharmaceutical industry. Once a model is built for a particular system, it becomes a powerful tool which provides insights into mechanisms and processes which may be experimentally unmeasurable, and to simulate outcomes under various conditions prior to or in lieu of conducting the actual experiments. Such an example would be to evaluate the impact of altering airplane wing characteristics on handling, speed, and fuel efficiency. With a valid mathematical model, the optimal wing can be designed for a given airplane without the need for extensive flight tests.

Mathematical modelling of the pharmacokinetics and pharmacodynamics of drug action, as illustrated in Figure 1, has been successfully applied in a number of settings [27,30]. Such models of drug action ideally relate the pharmacokinetics and pharmacodynamics to clinical outcomes of both safety and efficacy. By incorporating the sources of PK and PD variabililty, it is possible to predict the clinical outcomes that a given dose administered to a large population of patients will have. This approach can be utilised to successfully simulate clinical trials, and assist in optimising clinical trial designs prior to study conduct. Depending on the performance of a particular model, clinical trial simulations based on a valid model may obviate the need to conduct certain clinical trials, and allow for more efficient and expedited regulatory filings in drug development.

PK/PD modelling incorporates variability in drug response across a population of individuals by statistically testing the contribution of various patient factors. Such factors, or variables, are called covariates. Covariates most commonly include patient demographics (such as age, race, body weight), concomitant medications, and concomitant disease states. For example, for a renally eliminated drug, it is expected that an individual patient’s renal function would be incorporated into the model as a covariate, as this will have an important impact on pharmacokinetics and drug exposures. Patients with reduced renal function will have reduced clearance, higher drug exposures, potentially increasing risk of toxicity. With the use of modelling, it is then possible to determine an appropriate dose of a drug which should be administered to a patient with renal impairment.

As genetic testing becomes more widely available, the results of these tests may be incorporated into mathematical models as additional covariates which contribute to the variability in drug response observed over a large population. Incorporating genetic test results in addition to traditional covariates may significantly improve the utility of mathematical modelling to individualise drug therapy. 

In order to achieve the goal of personalised medicine, to deliver optimal individualised therapy, model based methods appear to be a promising approach. A particular model, built at the population level, may incorporate as much pre-existing information to get the best “first guess” as to the best drug and ideal dose of a particular medication to a given patient at the start of therapy. This baseline information might include demographics, concomitant medicines, disease-state markers, and the results of key genetic tests. In this case, the model can be used to select an initial drug and dose which will provide the highest probability of a successful outcome. After starting treatment, measures of drug response for safety and efficacy in the individual patient can be taken to individualise the model. As more information is collected, the model becomes increasingly precise at predicting the optimal treatment regimen for an individual patient. 

There are a number of successful illustrations of the use of model based methods to individualise therapy [31,32,33,34,35,36,37,38]. One example relates to the individualisation of carboplatin treatment based on thrombocytopenia. A mathematical model of carboplatin effects on platelets was developed. As thrombocytopenia is the dose limiting toxicity of carboplatin, the goal was to individualise the maximum tolerated dose (MRD), rather than using a population derived MTD defined in Phase I trials, which provides an MTD which is too low for some patients, and too high for others [37]. The concept was that clinical outcomes in some patients who are less sensitive to carboplatin-induced thrombocytopenia could be improved from receiving higher doses. Carboplatin pharmacokinetics are highly correlated with a patient’s renal function, and measurement of carboplatin concentrations in the individual patient is not necessary [39]; the desired exposure (AUC, area under the plasma concentration curve) can be obtained by measuring renal function. Patients were administered a standard AUC of carboplatin, and following this dose the impact on platelets was modelled to derive individualised model parameters. Based on the response to the first dose, the second dose administered several weeks later, could be determined based on platelet response. This was repeated in an iterative manner, until an individualised MTD of carboplatin was identified. Patients dosed using this model based approach received a significantly higher total carboplatin dosage compared to the standard approach, which may potentially result in improved clinical outcomes. 

There have been several important barriers restricting the widespread uptake of individualised model based approaches to optimize therapy. These limitations include (a) knowledge and ability of practitioners to work with mathematical models and availability of technology, (b) lack of drug assays for real-time measurement of pharmacokinetics, and (c) regulatory acceptance of such approaches. 

To be successful, model based methods must be both easy to use in practice and readily available to health care providers. The rapidly growing availability of web-enabled devices and databases may significantly improve the ease of use of such methods. Whereby previous complex algorithms would need special software and dedicated computers run by a knowledgeable individual, it is now possible that such algorithms could be run through a handheld phone application or via the internet and pushed to a device. The necessary data could be easily captured in electronic medical records, or through the device itself. As patients become more empowered in the future healthcare system, it is not difficult to see such applications being run by patients, with pharmacodynamic data collected by patients (such as blood glucose levels, blood pressure, or other novel home tests), fit by an individualised dosing model, and transmitted via the internet to the physicians computer record for rapid and efficient modification of therapy. Such algorithms would incorporate all relevant model covariates, genetic and non-genetic in nature. As patient response databases are developed, linking disease factors and outcomes, the model would continuously be refined and become better and more efficient over time at rapidly optimising therapy in the individual patient.

## 6. Implications for Drug Development

For drugs with only little response variability there will be little impact since a single dose level will be equally effective, safe and cost-effective in all subjects. Likewise for drugs expected to have wide therapeutic indices where response variability can be easily compensated for by using a relatively high, but safe, dose in all subjects. 

Of course there is always the possibility that such drugs may have unexpected adverse effects and therefore either be limited to lower doses or require some means of being able to identify the subjects likely to have the best or worst risk : benefit ratios. Clearly the earlier such adverse effects are detected the better, but even if they are identified late in development or after approval it is still possible to develop tests to stratify the patient population and identify groups in whom the drug should be avoided or in whom it is still appropriate to dose. The clinical utility of the drug is limited until sensitive and specific predictors (genetic or non-genetic) of adverse effects are determined but once available the drug can be used more widely. The association of abacavir hypersensitivity with HLAB57*01 exemplifies the advantages of such stratification whilst the lack of a predictive test for clozapine agranulocytosis has relegated it to use only in patients who have not responded well to other anti-psychotics.

However, the mechanism based or otherwise dose-related adverse effects must be identified early in development in order to develop effective compensation. The need would be to stratify patients to different doses and such dose stratification would have to be studied in a clinical trial program to demonstrate safety and efficacy of the different doses in different patient populations. It is possible that non-response to clopidogrel could be overcome by increasing the dose in non-responders. Since the primary adverse effects are mechanism-based this would be expected to be safe as well as increasing efficacy in as many as a third of all patients. Platelet aggregometry is a plausible biomarker of effect but the exact relationship between inhibition of platelet aggregation and clinical efficacy is unknown so the use of higher doses in non-responders could only be recommended once the appropriate clinical trials have been performed to confirm efficacy and safety in this subgroup. 

Likewise, for more expensive drugs or those entering relatively crowded markets, it is important to identify response or dose predictors early in development. Clopidogrel has been a highly effective drug despite the issue of non-response in a significant minority of patients. However, until recently, it had no significant competition, was relatively cheap and clearly cost effective. For expensive drugs or those in a competitive market place the responder populations or those needing different doses must be identified early and the trial program adapted to accommodate them. This was clearly of value in the development of omalizumab, as described earlier, whereas failure to identify the predictive value of k-ras mutation in tumour biopsy as a non-response predictor significantly decreased the clinical utility of early anti-EGFR antibodies and there was limited clinical uptake due to expense and apparently limited clinical benefit. As the demand grows for more and more cost-effective drugs it can be expected that the need for response predictors and/or dose stratification and optimisation will be increasingly important to ensure clinical benefit in the majority of patients. It will be important to explore and understand response variability as early as possible during drug development and to study a wide range of potential response predictors, both genotypic and phenotypic in order to maximise the chances of developing effective strategies for patient and dose stratification to include prospectively in subsequent clinical development. In some cases novel tests will need to be developed to permit response prediction. Ideally such prognostic tests will need to be developed and used during clinical development to demonstrate their predictive value and clinical utility. In other cases the response predictors will be tests that are already available in clinical use, or even be simple patient demographic characteristics, which can easily be accommodated into the development programme to confirm their value for stratifying or personalising therapy.
